# DeepNet model empowered cuckoo search algorithm for the effective identification of lung cancer nodules

**DOI:** 10.3389/fmedt.2023.1157919

**Published:** 2023-09-11

**Authors:** Grace John M, Baskar S

**Affiliations:** Department of Electronics and Communication, Karpagam Academy of Higher Education, Coimbatore, India

**Keywords:** cuckoo search algorithm, deep learning, encoder, jaccard similarity score, lung nodule

## Abstract

**Introduction:**

Globally, lung cancer is a highly harmful type of cancer. An efficient diagnosis system can enable pathologists to recognize the type and nature of lung nodules and the mode of therapy to increase the patient's chance of survival. Hence, implementing an automatic and reliable system to segment lung nodules from a computed tomography (CT) image is useful in the medical industry.

**Methods:**

This study develops a novel fully convolutional deep neural network (hereafter called DeepNet) model for segmenting lung nodules from CT scans. This model includes an encoder/decoder network that achieves pixel-wise image segmentation. The encoder network exploits a Visual Geometry Group (VGG-19) model as a base architecture, while the decoder network exploits 16 upsampling and deconvolution modules. The encoder used in this model has a very flexible structural design that can be modified and trained for any resolution based on the size of input scans. The decoder network upsamples and maps the low-resolution attributes of the encoder. Thus, there is a considerable drop in the number of variables used for the learning process as the network recycles the pooling indices of the encoder for segmentation. The Thresholding method and the cuckoo search algorithm determines the most useful features when categorizing cancer nodules.

**Results and discussion:**

The effectiveness of the intended DeepNet model is cautiously assessed on the real-world database known as The Cancer Imaging Archive (TCIA) dataset and its effectiveness is demonstrated by comparing its representation with some other modern segmentation models in terms of selected performance measures. The empirical analysis reveals that DeepNet significantly outperforms other prevalent segmentation algorithms with 0.962 ± 0.023% of volume error, 0.968 ± 0.011 of dice similarity coefficient, 0.856 ± 0.011 of Jaccard similarity index, and 0.045 ± 0.005s average processing time.

## 1. Introduction

Lung cancer is the most lethal cancer affecting men and women, leading to 18.4% of cancer mortality globally in 2018 (1). Current oncology breakthroughs including tyrosine kinase inhibitors and immune checkpoint inhibitors provide considerably larger survival improvements for cancer patients. But, much work is still to be carried out in computer-aided diagnosis systems, particularly in medical screening and timely lung cancer diagnosis. Automatic segmentation and classification would directly impact the workflow of medical practice in radiation oncology, one of the most widely used treatment methods for lung cancer (2).

Lung cancer radio therapeutics exploit medical imaging to find the exact location of nodules and electron densities to estimate the dosage at any point in the patient for disease management (3). Effective isolation of the lung nodules is indispensable as inaccuracies might cause under- or over-radiation of the malignant and benign cells. It is projected that a 1 mm variation of the nodule isolation could result in the radiation therapy dosage estimations by up to 15% (4). Hence, automatic and precise isolation can considerably decrease the time required for oncologists to plan effective therapy and re-plan adaptive therapy based on the variations in the nodules.

Manual segmentation of the lung nodules from CT scans is an error-prone and challenging task. As the correctness of the cancer diagnostic system depends on the severity of cancer and the oncologist's knowledge based on experience and decision, it leads to an inaccurate assessment (4). Due to lung nodules' very complex biological, molecular, and structural features, the analysis of these scans is hard in clinical pathology. Simultaneously, these confusing aspects encouraged numerous investigators to design new screening methods and analytic data to support the early detection of liver cancer, which can classify cancer cells with better accuracy and enhance clinical results. Besides, lung nodules' automatic screening will be faster than manual segmentation.

In conjunction with advances in storing and the quality of clinical scans, the current innovation of machine learning approaches has powered rigorous exploration in the domain of artificial intelligence (AI) for analyzing clinical images. Deep learning (DL) approaches are a section of AI-based neural networks that are effectively employed to resolve problems of image segmentation or classification at remarkable speeds without degrading accuracy (5). Many effective automatic diagnostic models have been established to solve these clinical imaging problems (6, 7). However, the main obstacle in designing a fully automatic tool is the inhomogeneity of the databases that can be used for any CT, particularly when collected from various medical organizations (8). CT images with various findings or reconstruction elements make the structure of the lung region different. The approaches found in the present literature frequently need powerful preprocessing methods. The data synchronization problem remains to be resolved by a data-oriented method, demanding large databases signifying all features of this heterogeneity.

The convolutional neural network (CNN) mimics the human visual system and is recognized to be the best image segmentation approach (9). Deep convolutional neural (DCNN) networks have recently been used to design an automated system for identifying and classifying lung cancer through medical imaging (10). This approach enables promising results, particularly in lung cancer segmentation. The usefulness of this approach on lung nodule segmentation has been evaluated against other machine learning methods recently (4, 7). Also, some researchers explored the benefits of exploiting deep convolutional networks for nodule segmentation against pathologists (11, 12). The decoder network makes use of 16 up sampling and deconvolution modules, the encoder network is predicated on a VGG model. This model employs an encoder with a highly adaptable architectural construction that can be learned to produce images of arbitrary resolutions, independent of the size of their input scans. In order to improve the encoder's low-resolution features, the decoder network up samples and maps them. The thresholding method removes redundant groups by isolating the particular lung regions and nodules of interest that the cuckoo search algorithm may conclusively identify. At the feature extraction stage, the surface highlighting for the specific nodule is unaffected by the instances from similar neighborhoods. DCNN outperforms oncologists’ decisions in nodule detection.

The main contribution of this paper:

i) This work proposes a fully convolutional deep neural network (DeepNet) model for segmenting lung nodules from CT scans.
ii) DeepNet model includes an encoder-decoder network to achieve pixel-wise image segmentation.
iii) The cuckoo search algorithm separates the lung nodule in the dataset images in the form of segmentation
iv) The encoder module exploits a VGG-19 model as a base architecture, whereas the decoder module exploits 16 upsampling and deconvolution modules.
v) The encoder has a flexible structural design that can be modified and trained for any resolution and size of input scans.
vi) The decoder module upsamples and maps the low-resolution attributes of the encoder. Thus, there is a considerable drop in the number of variables used for the learning process as the network reuses the pooling parameters of the encoder for segmentation.
vii) The DeepNet has been evaluated in terms of error rate, dice similarity coefficient, Jaccard similarity index, and average processing time.

The remaining parts of this manuscript are arranged as follows: We explore the related works about DCNN-based nodule isolation methods in section 2. Section 3 discusses the segmentation network and explores how every phase operates. Section 4 discusses the experimental procedure used in this work. Finally, we conclude Section 5.

## 2. Related work

Recently, numerous DL methods have been developed to solve the segmentation problem. In this section, we have studied some DL methods for handling lung cancer isolation. Mukherjee et al. developed an isolation approach using DL to localize the lung cancer and retain the nodules' structural characteristics through the graph cut technique (13). The outcome of isolation approach is77.67% of DSC and ASD of 0.24. The proposed model is not trained for comprehensive dataset and need to investigate how increasing the model's network complexity can impact its effectiveness.

Wang et al. developed a multiple-view convolutional network to segment lung cancer by collecting axial, sagittal, and coronal observations about the cancer voxel (14). The outcome of multiple-view convolutional network is77.67% of DSC and ASD of 0.24. The proposed model is not trained for comprehensive dataset and need to investigate how increasing the model's network complexity can impact its effectiveness.

Roy et al. proposed a technique to integrate a level set and DL for isolating the affected regions in the lung (15). Since the lung is a three-dimensional (3D) structure, we need to review 3D DL models for segmenting lung nodules. More accuracy is produced for the proposed method. The segmentation of lung nodule is the major drawback.

Hossain et al. proposed a novel dilated hybrid-3D CNN structure for nodule segmentation (3D LungNet). It can exploit the 3D data existing within image volumes. First, a binary classification algorithm selects images that may include slices of a nodule (16). To isolate the nodules, the designated images are fed to the DL algorithm for segmentation which selects attribute vectors from every 2D slice through dilated CNN and then combines the pooled vectors using 3D convolutional operations by integrating the morphological features in the CT image. The automated pipeline for lung tumor detection outperforms all segmentation network. Plans for the future include simultaneously developing a binary classifier and segmentation system, as well as training the pipeline with thicker stacks of segments.

Badrinarayanan et al. proposed a new deep CNN structure for semantic element-wise isolation called SegNet (17). This primary isolation model contains an encoding module, an equivalent decoding module, and an element-wise classifier. The role of the decoder module is to relate the lower-resolution attribute vector of the encoding module to the high-resolution input attribute vector for element-wise analysis. The innovation of this model lies in how the decoding module unspools its distorted input attribute vector. Especially the decoding module exploits pooling parameters calculated in the corresponding upsampling phase to achieve non-linear unspooling. This removes the training predictability of unspooling operations. SegNet has competitive performance on big and datasets, including strong scores for roadway scene interpretation. The end-to-end learning of deep division structures deserves more research effort because it is a more difficult topic.

Chen et al. propose a new 3D DL model for lung nodule isolation from CT scans, called multiple-attention U-Net (MAU-Net) (18). This model first uses a dual attention unit at the restriction of the U-Net that defines the definite relationship between channel and spatial attributes. The multiple-attention unit is then used to dynamically compute and combine multiresolution attributes from the dual attention unit from the encoding module. ResNet exploits different residual convolutional modules to extract the CT scans' significant attributes effectively. The attributes from all levels of the ResNet were combined into a single output. This simple architecture realized a combination of shallow appearance attributes and deep semantic attributes to produce dense pixel outputs. The lung cancer segmentation by existing dual attention methods is well explained. The proposed methods fail to investigate its performance in various medical imaging tasks.

Zhao et al. developed a contextual CNN using 3D U-Net to isolate and categorize nodules automatically and help oncologists interpret CT scans (19). The skip connections in conventional U-Net cause distortion in selected input attributes. On the other hand, the higher-resolution attributes selected by this model generally do not comprise sufficient higher-level boundary statistics of the image, causing distress in the decisions of the model significantly. Finally, in order to decrease the number of false positive candidate nodules, a contextual CNN is utilized to categorize nodules as malignant or benign. The main advantage is that contextual information of nodule is considered for the prediction process. The proposed method need to be considered for small dataset.

Seo et al. developed a Modified U-Net (mU-Net) for segmenting lung cancer from CT scans (20). This model exploits a residual unit with de-convolutional and activation functions using dropout connections to resolve the issues due to low-resolution attributes in conventional U-Net architecture. U-Net is a widely recognized CNN model for isolating lung nodules. The U-Net structure contains two parts; a shrinking phase to collect background information and a symmetric growing phase to achieve precise localization. The shrinking phase comprises successive convolution and max-pooling layers. It is employed to select features while limiting the size of the feature vector. The growing phase contains convolution layers and achieves up-conversion to obtain the attribute's dimension related to the loss of morphological features. Besides, the localization data is exchanged between the shrinking and the growing module using dropout units. These connections are operated autonomously and permit information to be communicated from one module to another within the network without adding any processing overhead. Finally, this research introduces a more powerful deep learning network for segmentation, which, depending on the specifics of the situation, may provide better outcomes than competing networks in the segmentation of liver and tumor areas, where the border is not evident and the target item is tiny.

Jalali et al. proposed an adapted U-Net where the encoding module is substituted by a learned ResNet-34 model (21). This network uses a bidirectional convolutional long short-term memory to integrate the selected attribute vector of the equivalent shrinking phase into the earlier growth of the up-convolution module. Then, a densely connected convolution module is used for the shrinking phase. Several abovementioned models have accomplished their goals effectively. However, their segmentation performance in terms of volume error, dice similarity coefficient, Jaccard similarity score, and average segmentation time is often not the best. Therefore, a novel model called DeepNet is developed for segmenting lung nodules with improved performance. The problem of a large number of false positives has also been addressed by the proposed strategy. The proposed strategy has also overcome the problem of removing nodules that have become connected to the lung wall. A further possibility for future efforts is the use of several deep learning-based systems to classify medical pictures.

Li et al. used liquid biopsy to provide a novel method for early screening, diagnosis, and management of lung cancer, particularly when tissue samples are unavailable (22). The use of circulation biomarkers and liquid biopsies in lung cancer allows for assessing the immediate molecular, genetic, and epigenetic profile of cancerous cells identified as drug-resistant clones from earlier therapy. Liquid biopsies are helpful because they are non-invasive, simple to collect, represent the overall condition of cancer, and provide real-time surveillance. Before liquid biopsy can be broadly employed in clinical practise, it needs to be improved through the employment of more cutting-edge molecular biological detection techniques in order to increase its validity and applicability.

Jiang et al. described the multimodality MRI-based radiomics approach for predicting lung cancer (23). Radiomics characters are derived using random forest techniques using the multimodal MRI scan data. After collecting data from the initial cohort, the randomized forest radiomics score varies depending on a separate group of individuals. The accuracy of the predictions was determined using a combination of the ROC curve, the calibration curve, and the decision curve.

Ji et al. designed a encoder-decoder design approach to normalize 3D point clouds by taking properties into account, before building voxels to feed into the procedure for learning (24). The suggested technique performs exceptionally well overall, making a significant contribution to the multi-class object identification from 3D tunnel clouds of points.

Amr Abdelraouf et al. modeled attention-based multi-encoder-decoders (Att-MED) for estimating travel times (25). Short-term, daily, and weekly traffic trends are only some of the input sequences that are used by the model's convolutional- Long short-term memory (LSTM) to record the spatial-temporal link between them. The model also uses an LSTM to progressively predict outputs. In addition, an attention method is employed to quantify the value of each traffic sequence's input into the final forecasts. When the suggested network architecture is trained from beginning to end, it outperforms baseline models in terms of forecasting accuracy.

Wei et al. examined the Lane Changing (LC) procedure and recommends LC segmentation and sampling approach that divides the procedure into four distinct phases (26). Using route data from all four LC phases, we validate the optimum attention-aided encoder-decoder model and subsequently use it to inform the creation of a heuristic network model. While doing so, the suggested heuristic network is linked to the Deep Neural Network (DNN) to forecast vehicle kinematics data. Finally, a combined cascade prediction model is formed by testing the heuristic network and DNN in succession; this model may execute a fine-grained LC specification on the basis of the prediction outcomes. The experimental findings demonstrate that the suggested cascade forecasting model can accurately anticipate the trajectory, velocity, acceleration, and steering angle of a vehicle over a long period of time and can provide a fine-grained LC characterization. Further theoretical investigation intelligent connected vehicles (ICVs) and connected autonomous vehicles (CAVs).may benefit from the presented prediction model.

Wang et al. isolated distinct cell populations from single-cell transcriptome profiles, a single-cell deep clustering model using a dual denoising autoencoder with bipartite graph ensemble clustering (scBGEDA) is presented (27). Data is first offered to be projected into a low-dimensional place using a single-cell dual denoising autoencoder network, which can then learn feature illustration through explicit simulation for denoising reconstruction loss.

Yu et al. discussed regarding a single-cell model-based deep graph embedding clustering (scTAG) technique uses a deep graph convolutional structure to acquire cell-cell topological descriptions and to identify cell clusters in real time (28). To train the low-dimensional latent participation, scTAG incorporates the zero-inflated negative binomial (ZINB) model into a topological adaptive graph convolutional autoencoder.

Overall, several methods showed promise in lung nodule identification and tracking. Detecting infrequently formalized cancer from large and diverse volumes of lung CT scan images with varying contour, size, and location, the existing method accurately distinguishes between vascular, solitary, pleural, and juxta-pleural adenomas, demonstrating rigorous methods and techniques applicable across distinct datasets.

The suggested work utilizes a DeepNet model to automatically extract the self-learned characteristics for lung cancer detection based on their malignant untrustworthiness. Classification accuracy, sensitivity, specificity, and fewer false positive rate are improved. The methods, including the specific tools and data sets used, are emphasized, and the findings are compared to previous research in the field.

## 3. DeepNet model for segmenting lung nodule

Segmentation of lung nodules on CT scans is imperative for cancer disease management like analysis, radiation therapy, and reaction calculation. This work presents a fully automatic DL-based lung nodule segmentation model that can manage different CT scans. The DeepNet includes an encoder/decoder network that achieves pixel-wise image segmentation. The encoder module contains a VGG-19 model as a base architecture, while the decoding module exploits 16 upsampling and deconvolution units. The encoder used in this model is trained for any resolution and size of input scans. The decoding network unspools and relates the lower-level attributes of the encoder. Thus, there is a considerable drop in the number of variables used for the learning process as the model reuses the pooling parameters of the encoder for isolating the nodules in the lung region. Before processing the CT scans we need to apply preprocessing techniques to improve the quality of the image. DeepNet model is evaluated based on a real-time database by comparing its presentation with similar advanced models concerning volume error, dice coefficient, Jaccard index, and speed.

It takes a significant amount of time and it is not always accurate for doctors to confirm lung cancer using CT scans. With the suggested technique, clinicians can detect lung nodules early and analyze their interior structure. As part of the contribution to various problems with diagnosing lung cancer, the Cuckoo search algorithm extracts the area of interest with a unique segmentation method that employs thresholding. Using the Cuckoo search algorithm, nodules of varying sizes and shapes may be precisely separated with a small number of parameters.

**Figure 1:**
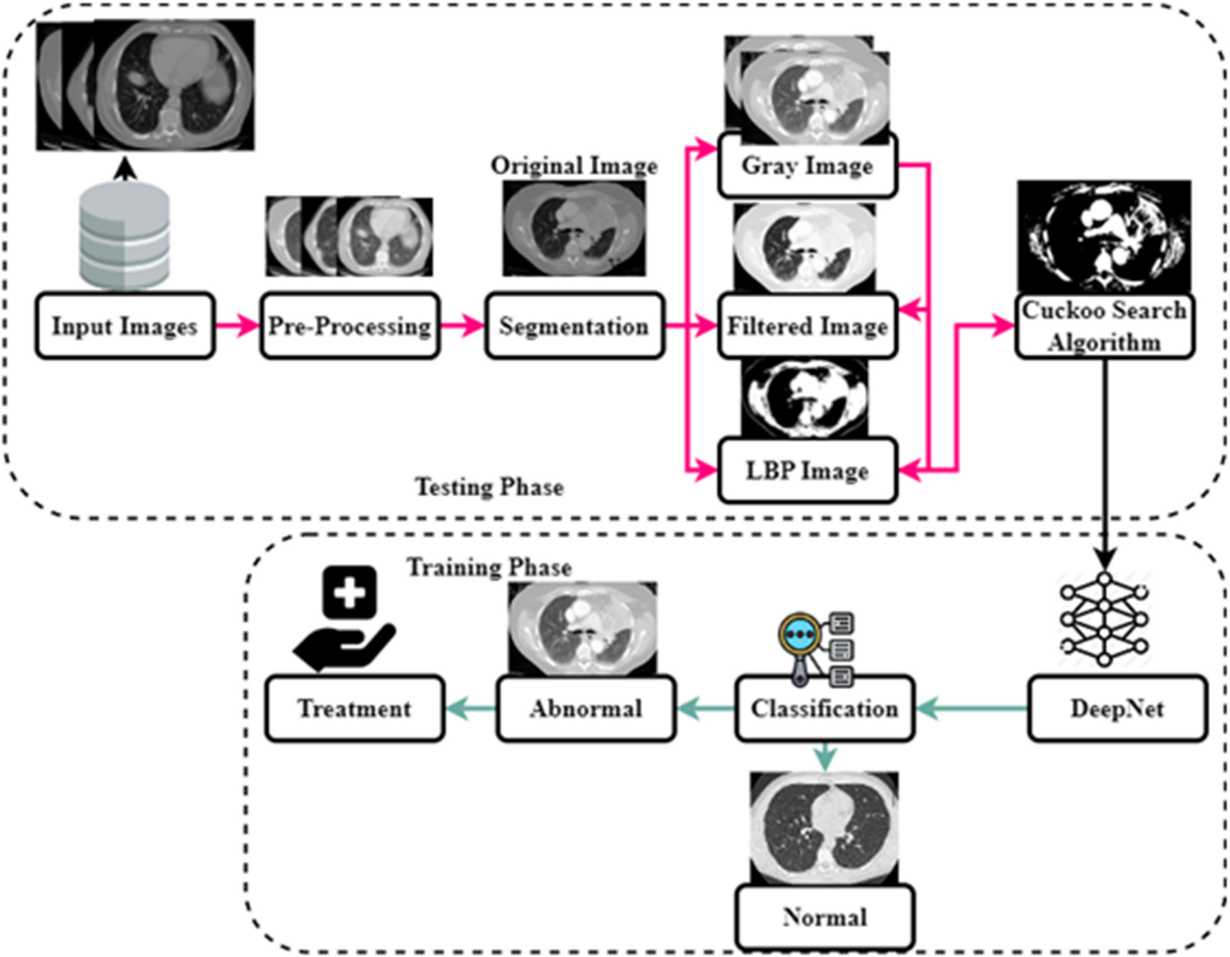
The architecture of the DeepNet model.

As shown in Figure 1, the steps involved in the future diagnosis of lung malignancy are as follows: (1) pre-processing to improve contrast and reduce noise; (2) using a Cuckoo search algorithm to separate the cancer cell from its surroundings; (3) feature extraction based on regions of concern; (4) retrieval of descriptive words from segmented lung lesions; and (5) using support vector machines to determine whether the injury is abnormal or not. Detailed explanations of these stages are provided below.

### 3.1. Image acquisition

It's the first step towards doing anything important. The technique involves retrieving and analyzing digital images from a source. A wide variety of scanners, including x-ray, MRI, and CT machines, are employed to get the final pictures. In this case, a CT scanner is used to get the picture. This scanning technique generates cross-sectional scans of each pixel. The obtained image is given for the next stage of pre-processing.

### 3.2. Data preprocessing

As the attributes are directly extracted from CT scans, it displays various gray scales and intensities essential to using a preprocessing strategy before such scans are fed to the segmentation algorithm. Data preprocessing approaches include normalization and standardization. This study employs a fast and simple method called a min-max scalar to achieve normalization as defined by Equation 1.

(1) $$\bar{\mu }=\frac{\mu -\mu_{\min}}{\mu_{\max}-\mu_{\min}}$$

**Figure 2:**
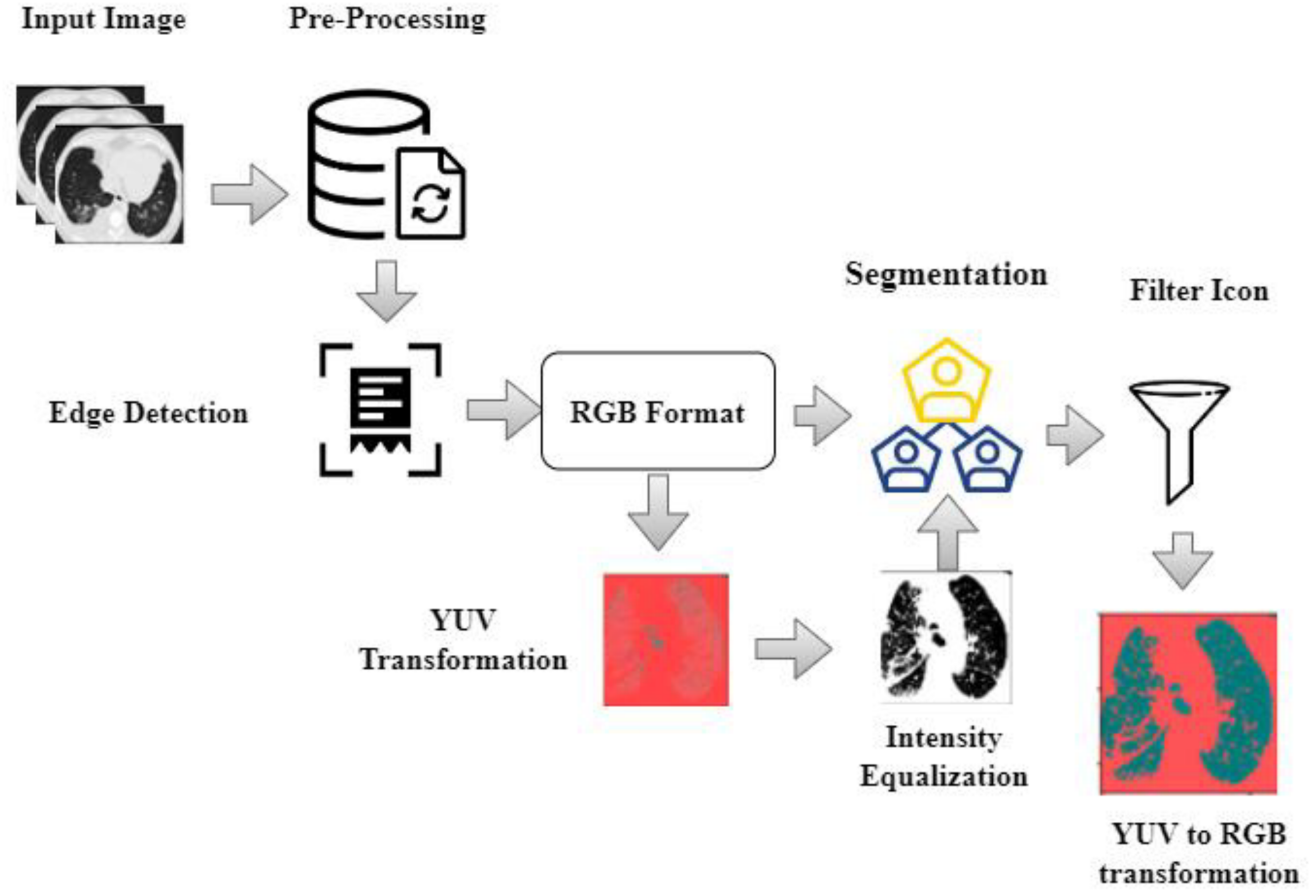
Pre-processed image and the output image as RGB transformation.

As shown in Equation 1 normalization has been expressed for the segmentation process. Where $\bar{\mu }$ is the normalized attribute value retrieved from attribute space μ, $\mu_{\min}$ is the minimum attribute value, and $\mu_{\max}$ is the maximum attribute value. In this paper, the input scans are preprocessed by eliminating noise and artifacts in the CT scans. Preprocessing includes the following phases: (i) the input scans are of various sizes and intensities. The input image with different sizes and intensities are used in the initial stage. The next stage is edge detection stages and this is obtained by grey scale images. The grey scale images are converted into RGB format luma and chroma. Luma and chroma color space is most significant for segmentation. Hence, all the scans have been transformed into a regular size of 128 × 128 before applying the segmentation algorithm; (ii) A filter with values ([−1, 0, −1], [0, 5, 0], [−1, 0, −1]) is used for finding edges of the nodules (iii) the values of each picture element are computed by transforming the red-green-blue (RGB) color to the luma and chroma (YUV) color space. Luminance is more imperative than color for segmentation. Hence, the resolution of V (red projection) and U (blue projection) are decreased however Y is preserved at high-resolution (iv) the intensity values of each picture element are balanced by transforming the YUV color back to RGB color space by flattening boundaries and intensity equalization. The pre-processed image is given to the DeepNet Nodule segmentation stage. In pre-processing stage the noise in image is removed using weighted histogram equalization and the intensity of equalization. From the pre-processed image edges are detected and the color image of RGB format is obtained. RGB image is transferred into YUV format. The above step is followed by segmentation and filtration. The final output image is obtained as RGB transformation as shown in Figure 2.

### 3.3. Nodule segmentation using DeepNet

**Figure 3:**
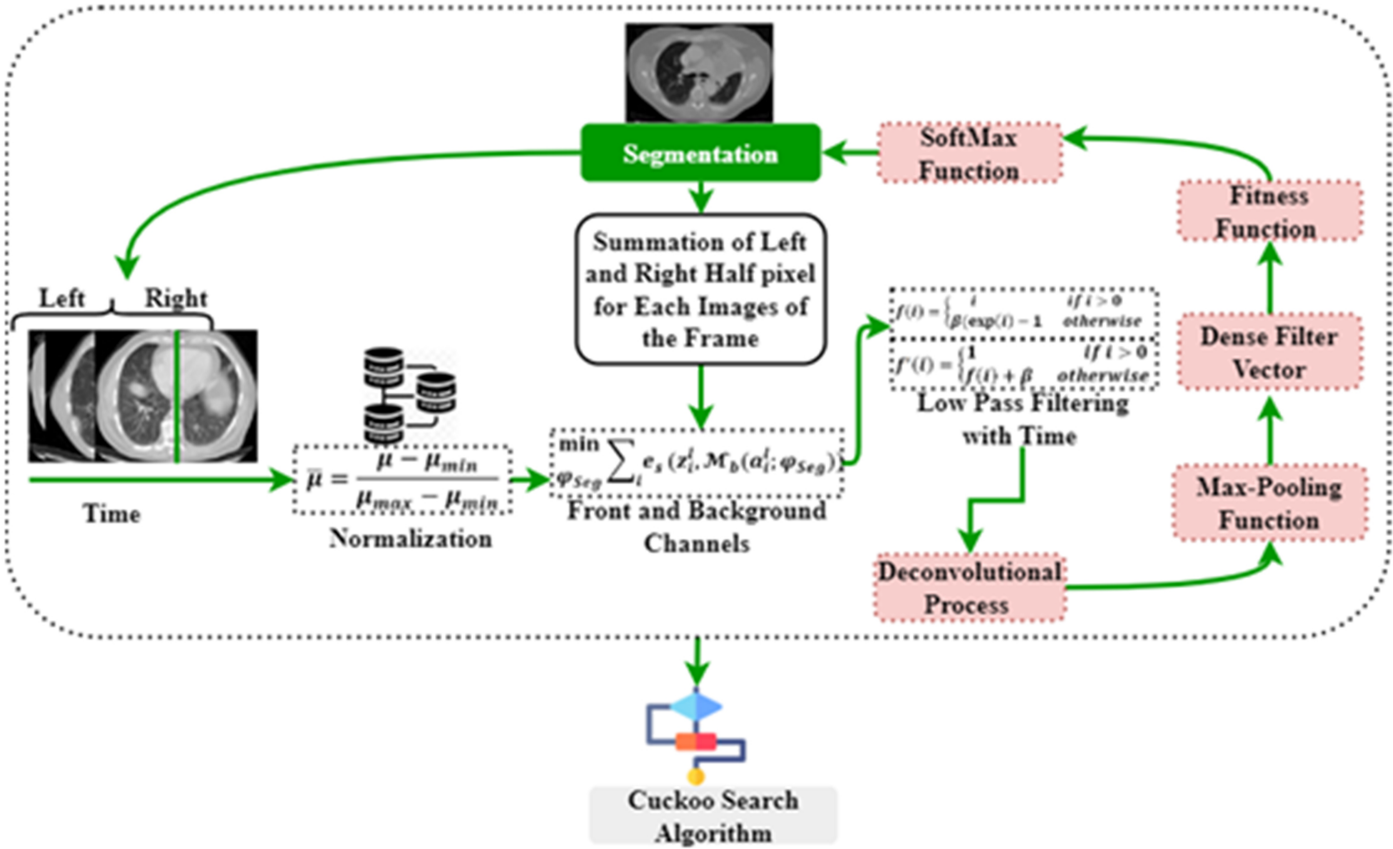
Path diagram of segmentation process.

This method seeks certain classes of pixels inside an image to create a concentrated image object and checks their proximity to one another. In medical imaging, segmenting the foreground data from the backdrop is challenging. The segmentation method excels at recognizing or smearing front objects' context information. Following the threshold collection standard, this method takes advantage of the largest class variation between the context and the target. The context represents the complete left and right section of the lung, whereas the target represent the affected region in the lung. It divides the picture into the foreground and background parts according to the tones of the grayscale. The separation between the two zones will be greatest if the smallest possible threshold is reached. Variance is a helpful measure, with a bigger number indicating a greater gap between the two regions. Separating the two is inadequate if the areas or contexts are incorrectly divided. Therefore, if there is a lot of variation across groups, the risk of making a mistake in classifying the cancer cell is reduced to get more continuous segmentation, as illustrated in Figure 3.

**Figure 4:**
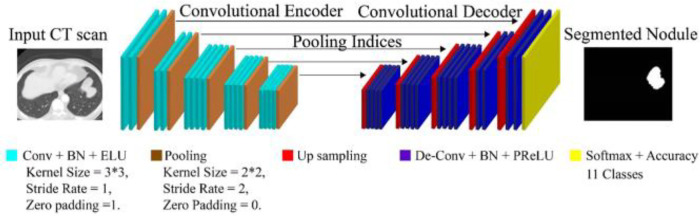
Architecture of DeepNet for lung nodule segmentation.

Figure 4 illustrates the architecture of DeepNet. It includes a pixel-wise semantic segmentation model with an encoder/decoder structure. The results obtained from the decoding module are given to a vital segmentation layer, which provides final nodule isolation. DeepNet uses the VGG-19 model as a base network. The fully-connected layers are dropped to preserve the maximum resolution attribute vector for the unspooling procedure in the decoding module. Thus, the size of the resultant pixel-wise encoding module is reduced related to other structures. In DeepNet, each encoding unit has convolution and batch normalization layers with a filter bank. These modules generate a set of attribute vectors. Then, the exponential linear unit (ELU) accelerates the training procedure.

The DeepNet model initiates with an input scan $S_{i}$ and activation map $\;a_{i}^{l}$. The input feeds data to the subsequent convolutional and pooling modules. From the attribute value calculation, the channel calculation is obtained from softmax segmentation. Finally, the decoding module is provided with an isolation vector $\;M(a_{i}^{l};\varphi_{\mathrm{Seg}})$, afterward applying the softmax (where $\varphi_{\mathrm{Seg}}$ is the segmentation parameter). $M(a_{i}^{l};\varphi_{\mathrm{Seg}})$, has the front and background channels. These channels are defined as $\;M_{b}(a_{i}^{l};\varphi_{\mathrm{Seg}})$, and $\;M_{f}(a_{i}^{l};\varphi_{\mathrm{Seg}})$, correspondingly.

(2) $$\begin{array}{c} \min\\ \varphi_{\mathrm{Seg}} \end{array}\underset{i}{\sum }e_{s}(z_{i}^{l},M_{b}(a_{i}^{l};\varphi_{\mathrm{Seg}}))$$

As deliberated in Equation 2, front and background channels have been described. Then, ELUs are used for activations to increase the speed of the training procedure. ELU converges to the maximum point at the end of the learning and validation procedures. The operation of ELU can be described as given in Equations 3, 4.

(3) $$f(i)=\{\begin{array}{cc} i & \;\mathrm{if}\;i>0\\ \beta (\exp(i)-1) & \;\mathrm{otherwise} \end{array}$$

(4) $${\;f}^{\prime }(i)=\{\begin{array}{cc} 1 & \;\mathrm{if}\;i>0\\ \;f(i)+\beta & \;\mathrm{otherwise} \end{array}$$

As found in Equations 3, 4 max-pooling function has been explored from ELU operation. Where β>0, the hyper-parameter is handled for the saturation of deconvolution layer inputs. The max-pooling modules are used with a stride rate of two and a 2 × 2 window. These max-pooling parameters are generated in the encoder and then unpooled in the decoding module. This approach keeps edge information in the isolated nodules and reduces training variables. The Batch Normalization (BN) module tests each convolutional module. Batch normalization data are computed from the learning procedure used for testing and skip sampling.

In DeepNet, a dropout method is used as an approximate inference to do probabilistic implications over the isolation process. The likelihood of dropout $\left(\rho_{i}\right)$ is optimized here. In our intended structure, the normal likelihood of skipping a connection is fixed at 50% (i.e., $\rho_{i}=0.5$). The first encoding module is provided with the equivalent (nearby) decoding unit with red-green-blue color space. This is different from the other encoder/decoder networks, which generate an equal number for the channel and size attribute vector based on the inputs given to the encoding module. As a final point, dense filter vectors are transmitted for pixel-wise isolation. The segmentation procedure is achieved using the softmax layer.

The fitness function of the DeepNet model is defined by Equation 5, and *i* values are correlated with the max-pooling function

(5) $$\zeta =\overset{N}{\underset{\;j=1}{\sum }}\overset{M}{\underset{i=1}{\sum }}-o_{j}^{\left(i\right)}\log s_{j}^{\left(i\right)}$$

As obtained in Equation 5 fitness function of the DeepNet model has been founded. Where *N* is the number of data samples and *M* is the number of the labeled class used in this study; $o_{j}^{\left(i\right)}=\{0,\ldots .0,\;1,\ldots 1,0,\ldots .0\}$ denotes the anticipated outcome vector; $s_{j}^{\left(i\right)}$ is the measured output vector of the *M* -th class. The classification (softmax) operation is given in Equation 6.

(6) $$s_{j}^{\left(i\right)}=\frac{e_{j}^{f}}{\sum_{i=1}^{M}e_{j}^{f}}$$

As demonstrated in Equation 6 softmax operation has been evaluated. Where $e_{j}^{f}$ is cross-entropy loss. Now, the function ζ can be adapted by the weight penalty to add a χ value to preserve the weights from getting larger in the fitness function of DSC, as given in Equation 7.

(7) $$\zeta =\overset{N}{\underset{\;j=1}{\sum }}\overset{M}{\underset{i=1}{\sum }}-o_{j}^{\left(i\right)}\log\left(\frac{e_{j}^{f}}{\sum_{i=1}^{M}e_{j}^{f}}\right)+\frac{1}{2}\chi \underset{K}{\sum }\underset{L}{\sum }\psi_{k,l}^{2}\;$$

As described in Equation 7 weights have been discussed. $o_{j}^{\left(i\right)}=\{0,\ldots .0,\;1,\ldots 1,0,\ldots .0\}$ denotes the anticipated outcome. Where $\psi_{l}$ denotes the link weight; KandL are the numbers of layers and connections in each layer, respectively. N represent the number of data samples and *M* is the number of the labeled class, $e_{j}^{f}$ denote the cross-entropy loss, j,i denote the number of outcomes $e_{j}^{f}\;$ represent the cross-entropy loss. *N*, *M* represent the number of samples and labeled classes. CSA follows the segmentation method.

### 3.4. Cuckoo search algorithm (CSA)

**Figure 5:**
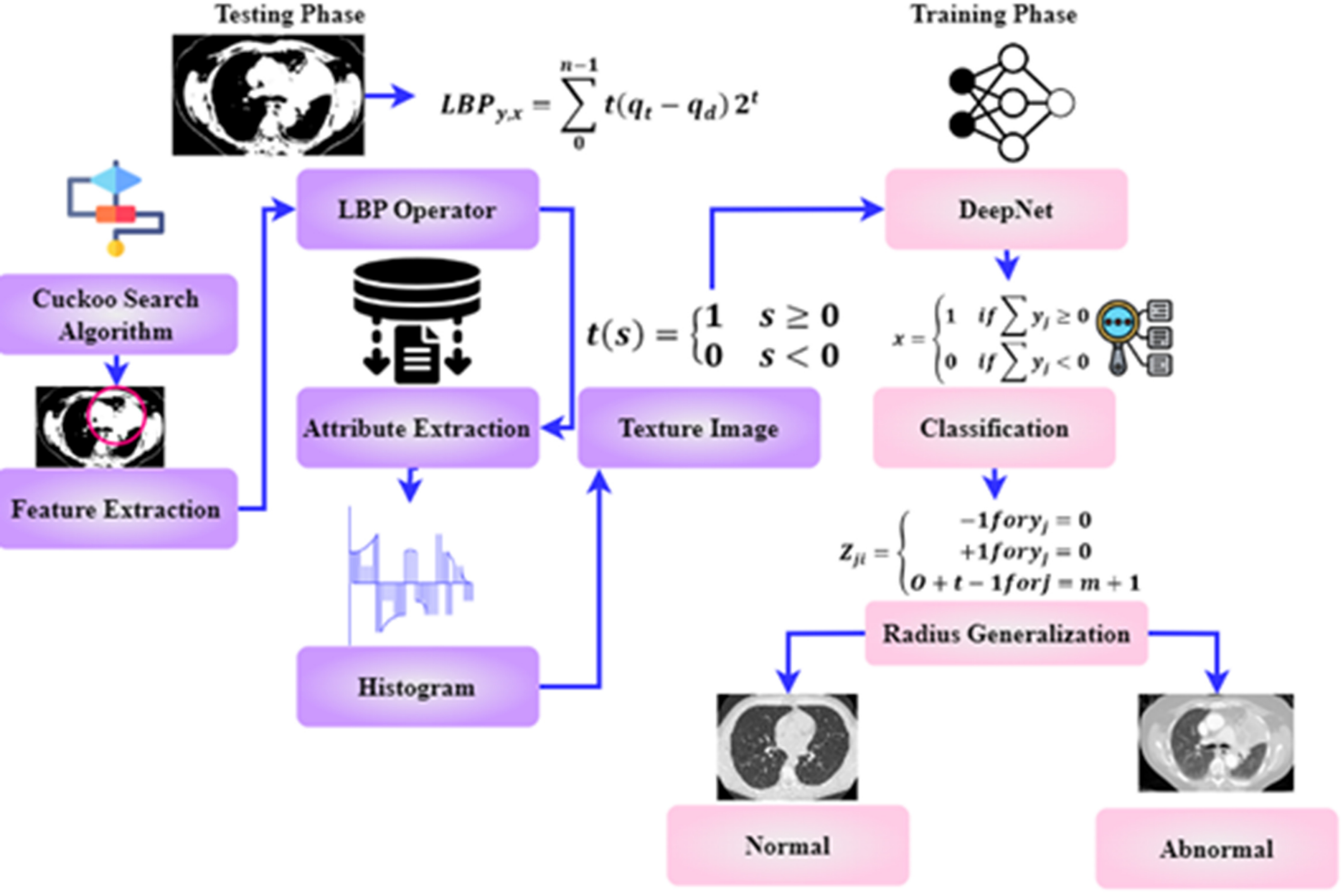
Path diagram of cuckoo search algorithm (CSA).

This method spreads the cuckoo generation function, reducing the situation's complications. During the search, the DeepNet method can reach many nests. A new solution has been found, which consists of discovering the position of the cuckoo egg. The following is an outline of the stages involved in the search operation. A cuckoo bird will lay one egg at a time in a nest that has been selected at random. The parasite nests did not change in any way, and the number of eggs inside them continued to rise until they reached their maximum capacity. When the cuckoo's egg is discovered, the bird hosting seems to have the option of either discarding the egg or destroying the nest and starting over with a fresh one. Figure 5 expresses the cuckoo search algorithm (CSA).

The CSA has been enhanced as a result of the Levy flight theory. CSA determines a suitable threshold for removing the lung nodule.The method that has been suggested for making the optimal selection (Algorithm 1) makes use of the analogy that is presented below:

**Algorithm 1:** CSA to divide up cancer

Input: *m*, Q&N
Output: $m_{j}^{s}$
If Q=1;
Return E
Else
$m_{i}^{s}=N+1$
End
For (*j* = 0; *j* > 0; *j*++)
$E=(m\_j^{s})$
End
If $m_{j}^{s+1}=m_{j}^{s}+\sigma ⊕\mathrm{le}v^{\prime }x(\delta )$
Return $x(\delta )=h^{-\delta }$
Else
$E=(m_{j}^{s+1})$
Choose $m_{i}$
End
If $\left(m_{j}^{s})>(m_{j}^{s+1}\right)$
Then
replace $m_{j}^{s}=m_{j}^{s+1}$
else
$m_{j}^{s}$
End

The new answer, which illustrates the subcategory of thresholds, is represented by an egg of a cuckoo. Additionally, CSA is exploited in the process of segmenting the lung nodule. The quality of the eggs each hosting nest produces is either 0 or 1, representing the threshold partition used in the segmentation technique. Pa is the likelihood that a cuckoo egg will be found by the bird serving as the host. Q has a threshold that has been established. This shows eliminating the boundary groupings that are least important to the overall study and deleting these threshold levels from further consideration.

The initial stage of the CSA algorithm is initiated with the input values *m*, *Q*&*N*. *m* is the number of hosting nests, *Q* is the likelihood of discovery of an alien, and *N* represents the highest amount of iterations. The generation of the initial host is given as $m_{i}^{s}=N+$. Then the for loop is executed for the *j* = 0 conditions. The evaluation of E is obtained from $E=(m\_j^{s})$. A new resolution is created for the resolution $m_{j}^{s+1}=m_{j}^{s}+\sigma ⊕\mathrm{le}v^{\prime }x(\delta )$ Where the symbol ⊕ is entry-wise multiplication, δ>0 indicates the step size, Lev $\;x(\delta )=h^{-\delta }(1<\delta \leq 3)$. Evaluate *E* $\left(m_{j}^{s+1}\right)$. Select a nest $m_{i}$ Randomly. If $\left(m_{j}^{s})>(m_{j}^{s+1}\right)$ then replace $m_{j}^{s}$ with $m_{j}^{s+1}$. Confiscate a worse nest with *q*. Create a new nest using Levy flights. Retain the best resolutions. From the segmented section of the cancer cell, the features are extracted in the following section.

### 3.5. Feature extraction

The original purpose of the LBP operator intended pattern detection. The operators label each pixel by thresholding the picture using the average pixel value and obtaining the resulting binary number. Attributes extraction consists of the following steps in the paragraphs below.

Make a grid on the window viewing through and combine all the pixels in a cell with their neighbors.

• At initial stage the value of “1” is assigned if the central pixel's value exceeds the adjacent pixel's and “0” otherwise.
• Then the binary integer may be generated by comparing each pixel with other pixels.
• Final stage is the histogram calculation for the whole cell
• The phrase allows one to determine an LBP value. Where *v* stands for uniform pattern and *y* and *x* are two numbers that have ${\mathrm{LBP}}_{y,x}^{v}$.
• Identify the surrounding area.
  (8) $$\begin{array}{c} \mathrm{LB}P_{y,x}=\overset{n-1}{\underset{0}{\sum }}t(q_{t}-q_{d})2^{t}\\ t(s)=\{\begin{array}{cc} 1 & s\geq 0\;\\ 0 & s<0 \end{array} \end{array}\}$$

As shown in Equation 8, the texture image has been deliberated. Where $q_{d}$ is the central pixel's grey value, $q_{t}$ is the modulation index of the position pixels (t=0,1,……t−1), and *n* is the pixel in the picture where *O* is greater than zero forming a locally-oriented neighborhood set. y,x are the numbers that covers an LBP value. The textured image is defined by creating a t(s) histogram after distinguishing each pixel in a photograph. The extracted characters are given to the classification stage for accurate classification.

### 3.6. Classification of lung images

DeepNet model recognition of lung cancer is the capstone of this study. A deep learning neural network does not manually extract the features for training; it may be used to prepare images for the classification process. Instead, deep learning uses the collected lung CT image or separated image to recognize the edges present in the picture and significant characteristics employed in the neural network training using a huge quantity of data. Due to the large dimensionality of the information, the network typically employs 150 hidden layers.

To forecast the manually extracted characteristics associated with lung cancer, the learned features are maintained in a database as a template. The procedure of classification involves self-training and categorization of the spectral characteristics that were manually collected. The network examines the lung characteristics and uses the extracted features in a manner consistent with the unstructured classification stage to the hidden node it has included. Particular network and weighting values are used to generalize the network and reduce the number of unnecessary characteristics required for classification. Since the network bias value is set to 1 during training, input nodes with moreover one of the total number of training features in the feature space are used. The relative importance of the connections in a network and the error reduction is represented here

(9) $$Z_{\;ji}=\{\begin{array}{c} -1\;\mathrm{for}\;y_{j}=0\\ +1\;\mathrm{for}\;y_{j}=0\\ O+t-1\;\mathrm{for}\;j=m+1 \end{array}\;$$

In Equation 9, *O* stands for the radius generalization, and t represents the hammering strength. The weight value from the hidden to output nodes is then specified as either −1or1, indicating that the feature is extracted from the lungs and is utilized to predict whether or not the features are a cancerous or noncancerous stage. At last, the value is disguised as output. The results of the training procedure are then characterized as follows,

(10) $$x=\{\begin{array}{cc} 1 & \mathrm{if}\sum y_{j}\geq 0\\ 0 & \mathrm{if}\sum y_{j}<0 \end{array}\;$$

**Figure 6:**
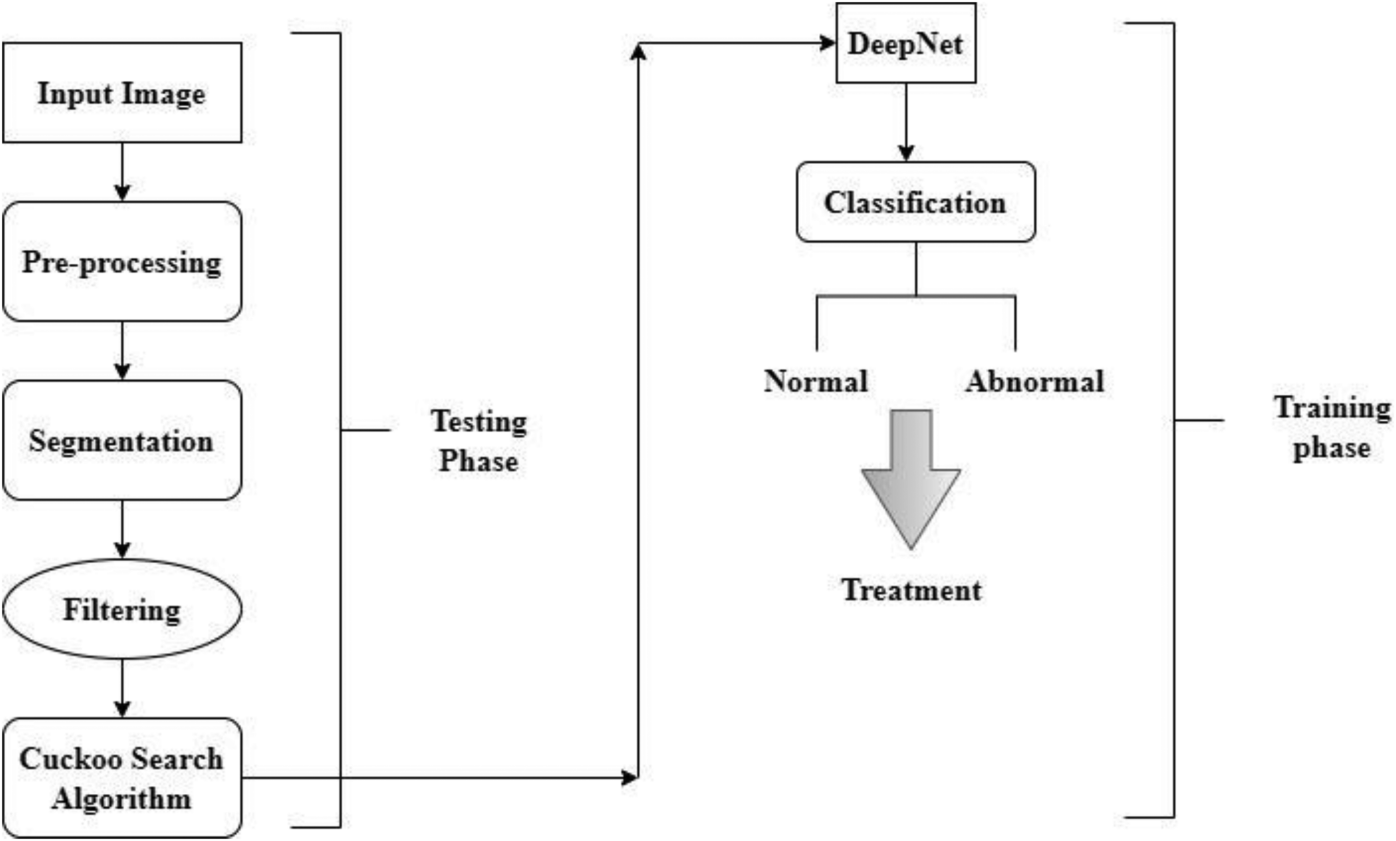
The flow chart of DeepNet model.

The obtained value has been compared to characteristics gathered and taught using deep learning for cancer classification. The classification of cancer cells is based on $y_{j}$ Characters as 0 and 1. The double-time comparison procedure increases identification accuracy and effectively decreases the false classification rate. The complete flow chart of the proposed system is shown in Figure 6. The input image is pre-processed, segmented and filtered. The filtered sample is given to Cuckoo search algorithm which is implemented in the testing phase. The training phase includes the DeepNet which is used for classifying the data sample as normal and abnormal. The treatment is implemented according to classification stages.

## 4. Empirical analysis

To demonstrate the effectiveness of DeepNet, a comprehensive experimental study is carried out on a 3.6 GHz Intel Core i7-4790 processor with 16GB memory and a Windows 10 operating system. The effectiveness of the DeepNet model is assessed by relating the results with six related classification models, viz. 3D LungNet (16), 3D U-Net (19), mU-Net (20), 3D-Segnet (17), MAU-Net (18), ResNet (21). All these networks including our DeepNet exploit deep learning algorithms for segmentation and are trained using the same training configuration. The comparison is illustrated in Root Mean Square error, F1-score Ratio, Efficiency ratio.

### 4.1. Dataset acquisition

To evaluate the effectiveness of any DL algorithm, we require a huge database that produces a better solution. This work uses a corpus of labeled lung CT scans from the TCIA database collected from the National Cancer Institute of Cancer Analysis Consortium Lung Cohort (29). The collected samples are related to proteomic, genomic, and medical statistics. The gathered scans are kept in the form of DICOM (digital imaging and communications in medicine) with definite labels including imaging, gender, date of birth, study dates, etc. 5,043 scans are used for segmenting lung cancer from various cases with 48 sequences. In this study, we purposefully divide the available database into 70% of instances (i.e., 3,530 images) for training and 30% (i.e., 1,513 images) of instances for testing.

**Figure 7:**
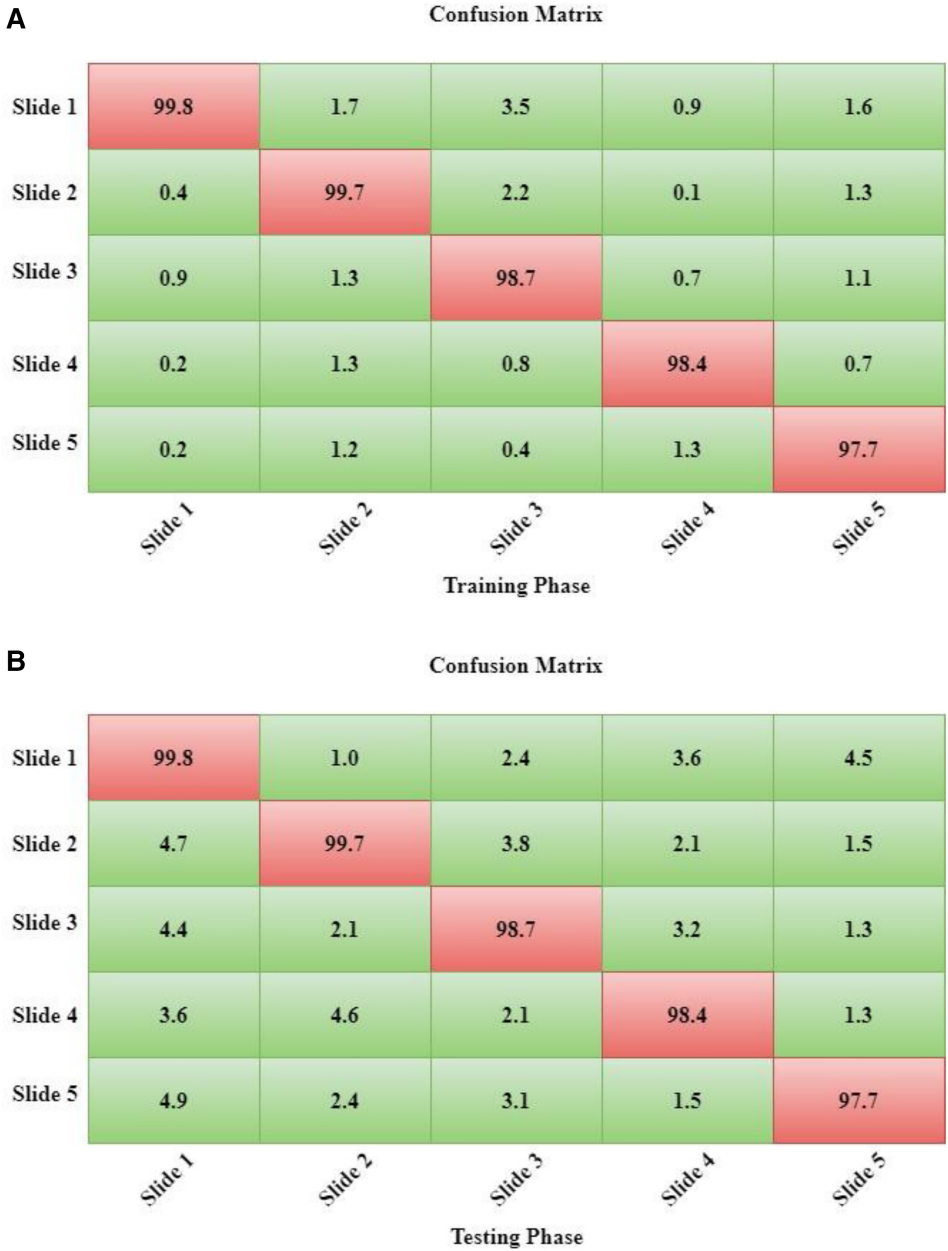
(**A**) the training phase of DeepNet. (**B**) The Testing Phase of DeepNet.

Moreover, since we employed 10-fold cross-validation (10-FCV), the total data samples are divided into 10 parts (each of 10%). Now, one fold (10%) is applied for testing, while the residual samples (90%) are split for validation and training. Using 10-FCV guarantees that each sample in the data gets to be in a test. 10-FCV is considered for five samples, and the confusion matrix for each sample is illustrated in Figure 7.

Dataset Description: DeepNet model aimed to develop a machine learning and deep learning (CNN) system to identify lung cancer. The main aim is to gather all information for quick and straightforward picture classification.

### 4.2. Performance measure

The performance measurement of DeepNet in the form of a confusion matrix is shown in Figures 7A,B. The calculation for both the testing and training phases of the confusion matrix is calculated for each samples. The sample data description is divided into 10 parts as five samples.

The performance of the DeepNet model is quantitatively assessed using some selected evaluation metrics like the volume error (VE), dice similarity coefficient (DSC), Jaccard similarity index, and average processing time. These metrics are computed by calculating the variation between the segmentation outcomes and a manually marked ground truth. The volume error is calculated using Equation 11.

(11) $$\mathrm{VE}=\frac{2\times (S-G)}{\left(S+G\right)}\;$$

where *G* is the ground truth (i.e., standard gold image) and S is the segmentation result obtained by DeepNet. For a medical practice, VE<5% is more likely acceptable (25). The Dice similarity score is generally employed to define the representation of the segmentation process on the input CT scan. DSC is a similarity index between two pixels. It refers to the fitness degree between the original and segmented images. The value of DSC is always in [0, 1] and is measured using Equation 12.

(12) $$\mathrm{DSC}\;=\frac{2\times |G\cap S|}{\left|G|+|S\right|}$$

The Jaccard similarity score (JSS) is an assessment measure employed to assess the effectiveness of any isolation method. Given a dataset, the JSS measure provides the similarity between the target scan and the ground truth. It is defined by Equation 10.

(13) $$\mathrm{JSS}\;=\frac{\left|G\cap S\right|}{\left|G\cup S\right|}\;$$

As explored in Equation 10 JSS has been demonstrated. This study considers the average processing time as the performance measure.

### 4.3. Empirical results

**Figure 8:**
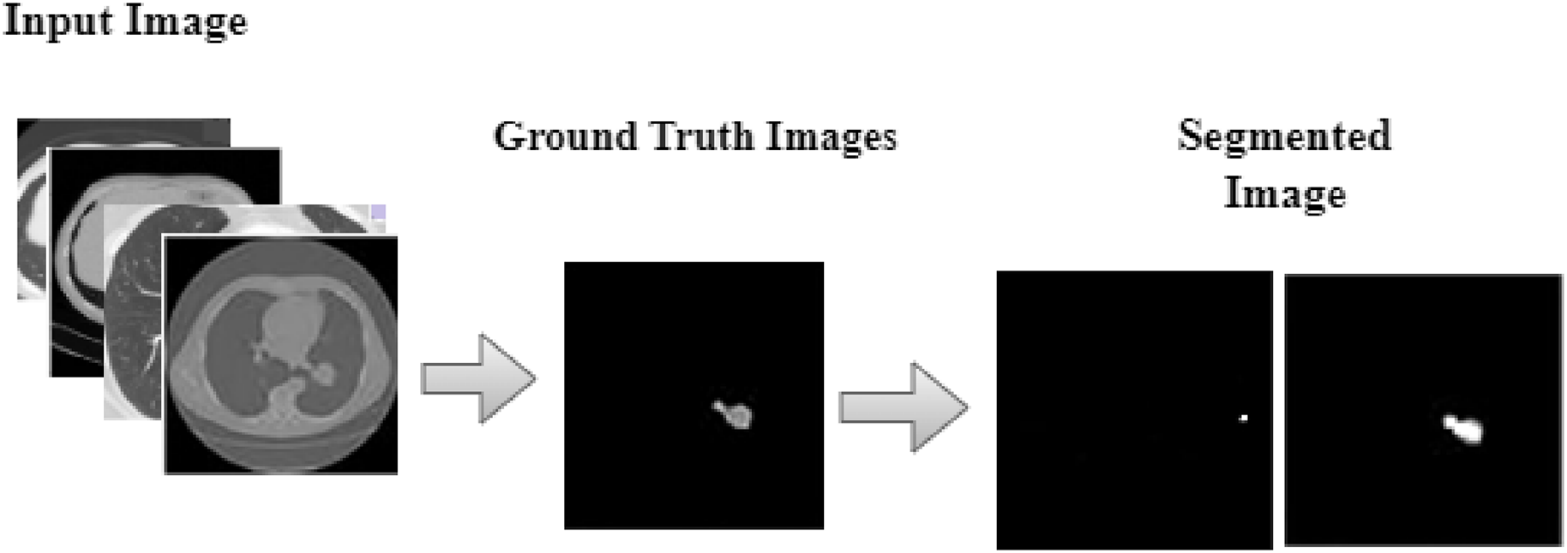
Segmentation results.

The DeepNet segmentation model is implemented using the deep learning toolbox in MATLAB R2018b software. The comprehensive outcomes obtained from the DeepNet segmentation model are given in Table 1. To realize more precise solutions, the 10-FCV technique is used. Consequently, the whole database is divided into 10 parts. For each trial, one part is applied for testing, and the other parts are used for training the classifier. Now the mean value of all ten tests is considered for evaluation. A comprehensive analysis of our results discloses the potential and weakness of our DeepNet approach. Generally, irrespective of the dimension of the standard gold image, the DeepNet model segments the nodules effectively. Figure 8 shows sample input images used for experimentation, segmented images obtained by DeepNet, and their corresponding ground truth images. Though the nodules are in different random localities within the lung and appear in various sizes, the segmented nodules coincide almost perfectly.

**Table 1:** The results obtained by DeepNet from the TCIA dataset.

Model	Criteria	VE (%)	DSC	JSS	Average processing time (s)
3D Lung Net	Mean ± std	4.871 ± 0.047	0.656 ± 0.154	0.687 ± 0.014	1.457 ± 0.002
3D Seg Net	Mean ± std	3.671 ± 0.041	0.786 ± 0.014	0.660 ± 0.012	0.083 ± 0.001
3D U-Net	Mean ± std	2.325 ± 0.059	0.843 ± 0.179	0.711 ± 0.011	0.160 ± 0.001
mU-Net	Mean ± std	1.347 ± 0.049	0.854 ± 0.183	0.758 ± 0.008	0.178 ± 0.002
MAU-Net	Mean ± std	1.145 ± 0.015	0.866 ± 0.133	0.804 ± 0.010	0.199 ± 0.003
ResNet	Mean ± std	0.345 ± 0.016	0.953 ± 0.014	0.814 ± 0.009	1.168 ± 0.004
DeepNet	Mean ± std	0.962 ± 0.023	0.968 ± 0.011	0.856 ± 0.011	0.045 ± 0.005

In classification process $T^{+}$ denotes the true positive, $T^{-}$ indicates the true negative, $F^{+}$ express the false positive, $F^{-}$ is examines the false negative.

Table 1 shows the isolation results obtained by the DeepNet model. The conventional 3D LungNet architecture provides 4.871 ± 0.047% volume error and 0.656 ± 0.154 DSC. 3D LungNet can exploit the 3D statistics present within CT scan volumes effectively. Therefore, this model can achieve a 0.687 ± 0.014 dice similarity coefficient and 1.457 ± 0.002 Jaccard similarity index. The average computational time per case of the 3D LungNet model is 1.457 ± 0.002 s. SegNet is more effective in terms of performance measures since it keeps the upsampling parameters of the attribute vector and employs them in its decoding module to realize better results than the 3D LungNet model. It provides a 3.671 ± 0.041 mean volume error, 0.786 ± 0.014 dice similarity index, and 0.660 ± 0.012 Jaccard similarity score. This model takes 0.083 ± 0.001 s for segmenting nodules.

The 3D U-Net approach achieves improved results compared to 3D LungNet and 3D SegNet models by concurrently applying the concept of context data and global position. Also, it guarantees the preservation of the entire quality of the input scans. Hence it achieves a relatively lower volume error (2.325 ± 0.059) and a higher dice coefficient (0.843 ± 0.179) as well as a higher Jaccard similarity index (0.711 ± 0.011). Moreover, it takes reduced mean processing time per case (0.160 ± 0.001 s). Conversely, higher-level attributes selected by this model often do not comprise sufficient higher-resolution boundary statistics of the input, leading to increased indecision where higher-resolution boundaries mainly impact the final decisions including the lung nodule segmentation.

The modified U-Net contains a residual block with de-convolutional and activation functions to the dropout unit to avoid distorted attributes' repetition. For small object inputs, attributes in the dropout units are not integrated with attributes in the residual block. Also, the recommended model has supplementary convolutional modules in the dropping out of a unit to select higher-resolution attributes of minor object inputs and higher-level attributes of higher-level boundary data of big object inputs. Hence, this network provides better performance for lung nodule segmentation in terms of dice similarity index (0.854 ± 0.183), the volume of error (1.347 ± 0.049%), and the Jaccard coefficient (0.758 ± 0.008). For effective nodule segmentation it consumes 0.178 ± 0.002 s for each sample.

By applying a 3D encoder/decoder-based CNN structure, MAU-Net realizes good performance for precisely isolating lung nodules from volumetric CT images. This model achieves performances of 1.145 ± 0.015%, 0.866 ± 0.133, 0.804 ± 0.010, and 0.199 ± 0.003 in volume error, dice coefficient, Jaccard similarity index, and average processing time, respectively. By applying the concept of dropout connections ResNet delivers 0.345 ± 0.016% of volume error, 0.953 ± 0.014 of dice similarity index, 0.814 ± 0.009 of Jaccard score, and 1.168 ± 0.004 s of average processing.

**Figure 9:**
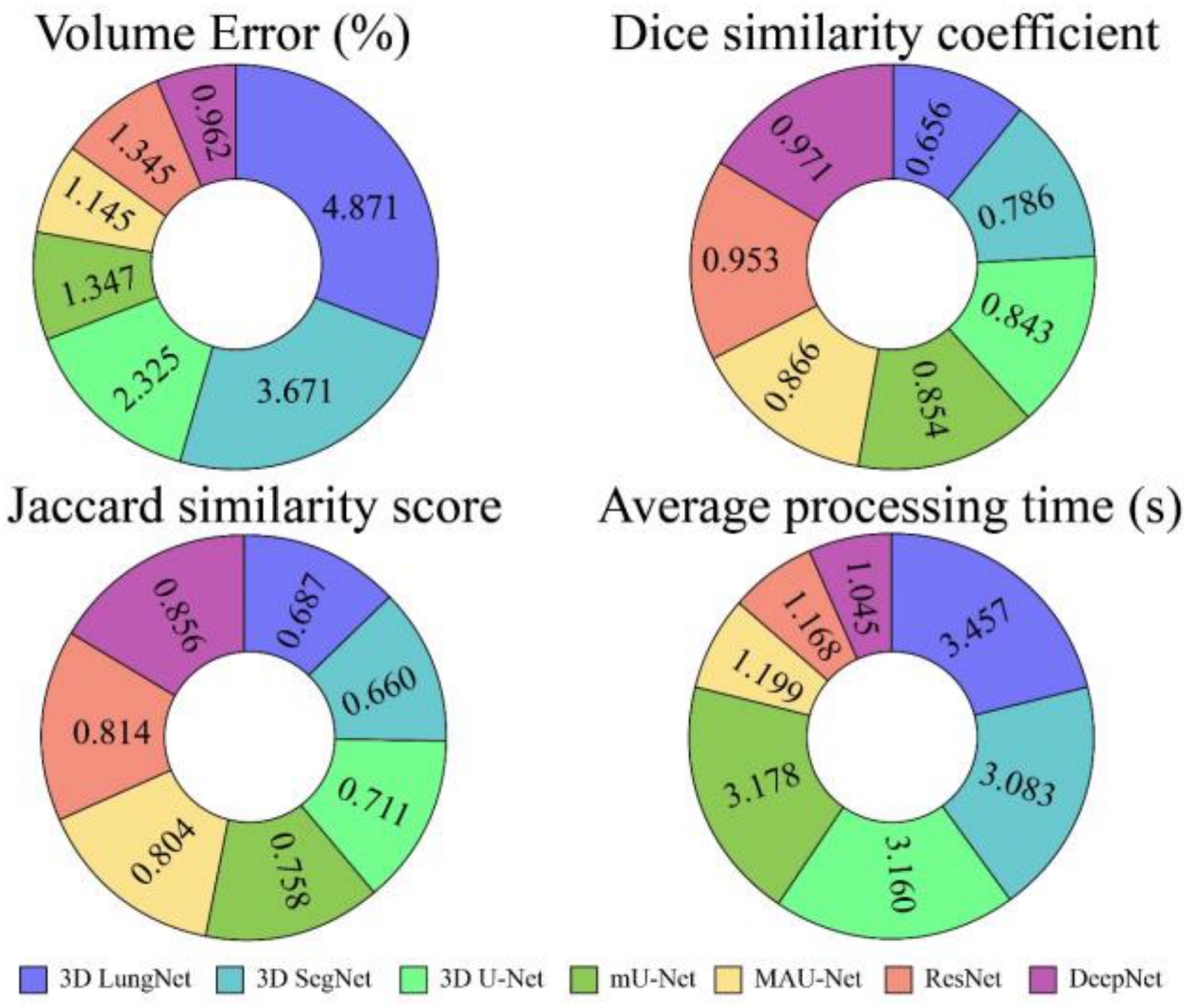
Performance of various segmentation models in terms of mean values.

**Figure 10:**
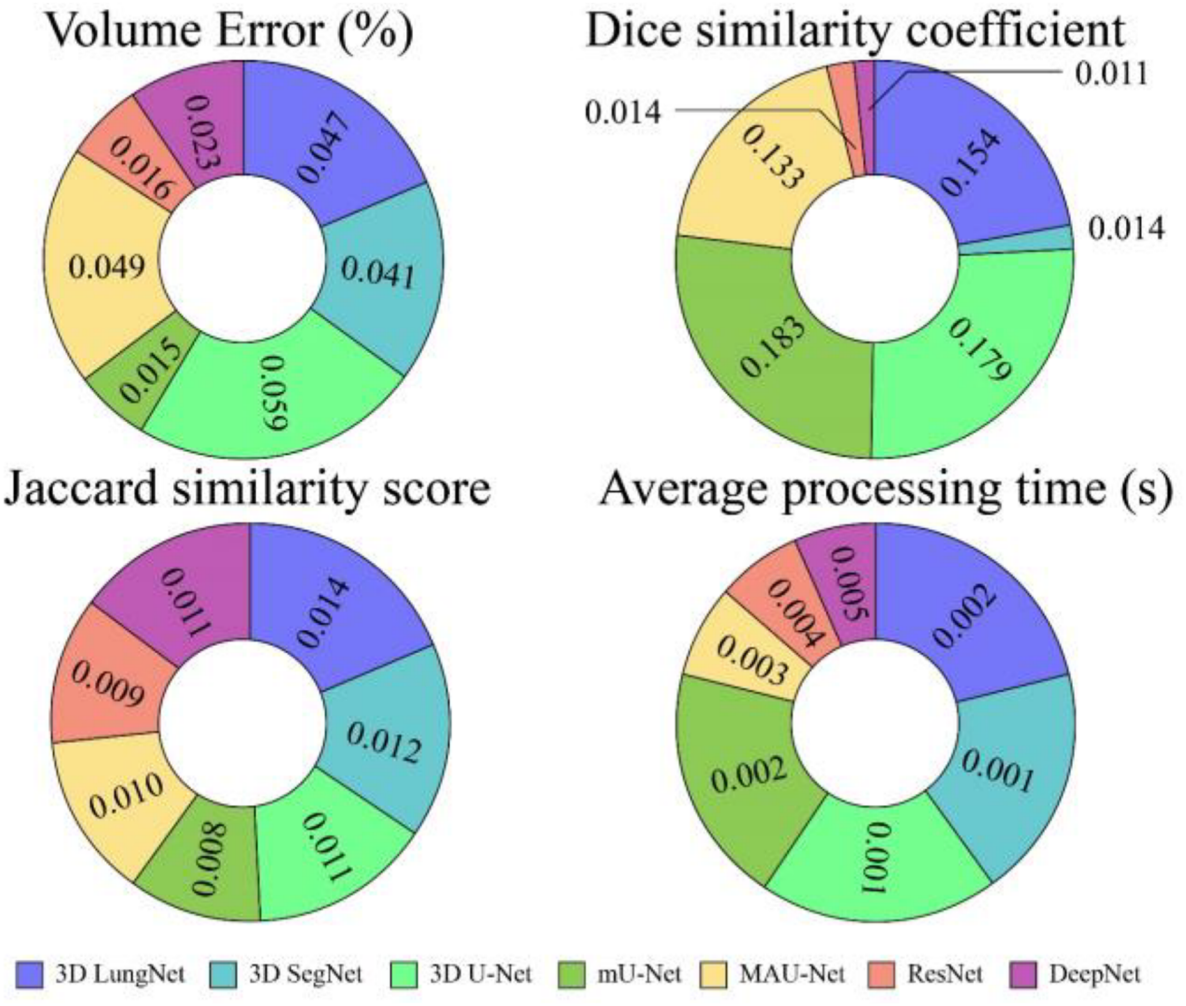
Performance of various segmentation models in terms of mean values.

The DeepNet model outperforms all other network models in terms of metrics selected for performance evaluation. DeepNet emphasizes that the attribute pooling function of DeepNet guarantees a less computationally expensive structure related to other segmentation models. It achieves performance measures of 0.962 ± 0.023, 0.968 ± 0.011, 0.856 ± 0.011, and 0.045 ± 0.005 in volume error, dice coefficient, Jaccard similarity index, and average processing time, respectively shown in Figures 9, 10.

Accuracy, loss, and computation time are all useful effective assessment characteristics that may be used to assess a medical image's efficacy. Regarding measuring the model's effectiveness, accuracy is a crucial metric. It returns the fraction of an image's pixels that have been properly labeled.

The neural network's error, or Loss, may be anticipated using the Loss function's computations. It's a key network performance metric. Computational Time is the amount of time it takes for a process to compute or carry out its actions. Time spent processing is reduced if the process is straightforward, whereas more time is needed for complicated procedures.

#### 4.3.1. Accuracy ratio (%) and loss curve

**Figure 11:**
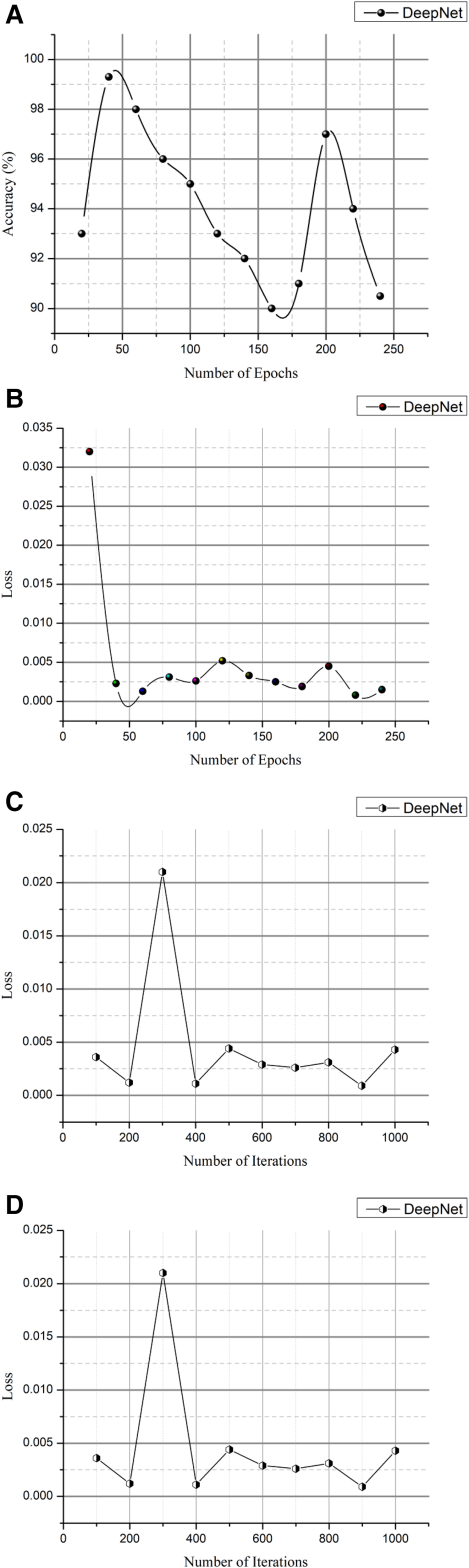
(**A**) Epoch vs. accuracy and (**B**) epoch vs. loss. (**C**) Iteration vs. Loss and (**D**) iteration vs. accuracy.

CT scans in DICOM format represent a total of 1,018 instances. Since it is challenging to train high-size pictures in DeepNet, the images were preprocessed to reduce the size to 512 × 512. Table 2 and Figures 11A,B show the epoch, loss, and accuracy results from the CT image dataset experiments.

**Table 2:** Epoch, verses loss, and accuracy results.

Epoch	Loss	Accuracy (%)
1	0.71	76.5
13	0.032	85.3
25	0.012	88.9
38	0.0045	90.1
50	0.0052	90.5
63	0.0033	91.5
75	0.0041	92
88	0.0025	92.5
100	0.0019	93
113	0.0026	93.5
125	0.0023	94
138	0.0015	94.5
150	0.0013	95
163	0.0016	95.6
175	0.0008	96
188	0.0056	97
200	0.0031	98
213	0.0013	98.5
225	0.0013	98.7
238	0.0013	99.1
250	0.0008	99.3

The loss and accuracy of the DeepNet for each epoch is shown in Figures 11A,B with the loss of 0.0008 and accuracy of 99.3. As shown in Equation 14, the classification accuracy rate is calculated

(14) $$Y=\frac{s}{m}\times 100\;$$

The total number of observations (*m*) is based on the particular number of accurate classifications (*s*). Training and testing images are separated into distinct categories in evaluating the network's efficiency in distinguishing cancer and non-cancerous pictures. The photos from the provided dataset are fed into the DeepNet model during training, with 90% of the images serving as examples. Table 3 and Figures 11C,D provide iteration, loss, and accuracy statistics from experiments performed on the CT image dataset.

**Table 3:** Iteration vs. Loss and Accuracy Outcomes.

Iteration	Loss	Accuracy (%)
1	0.71	73.5
50	0.022	82.3
100	0.021	87.9
150	0.0032	90.2
200	0.0044	91.6
250	0.0011	92.3
300	0.0021	92
350	0.0055	92.5
400	0.0029	94
450	0.0024	94.5
500	0.0026	94.8
550	0.0011	94.9
600	0.0012	95
650	0.0013	95.1
700	0.0007	95.8
750	0.0036	96
800	0.0031	96.4
850	0.0043	97.5
900	0.0033	97.7
950	0.0023	98.1
1,000	0.0009	99.5

The loss and accuracy of the DeepNet for each Iteration is shown in Figures 11C,D with the loss of 0.0007 and accuracy of 99.5. After the training phase is complete, 10% of the same dataset's testing images are used to assess the model's performance. Here, the samples of photos are fed into a network model for cancer/non-cancer image classification.

#### 4.3.2. Computation time (%)

**Figure 12:**
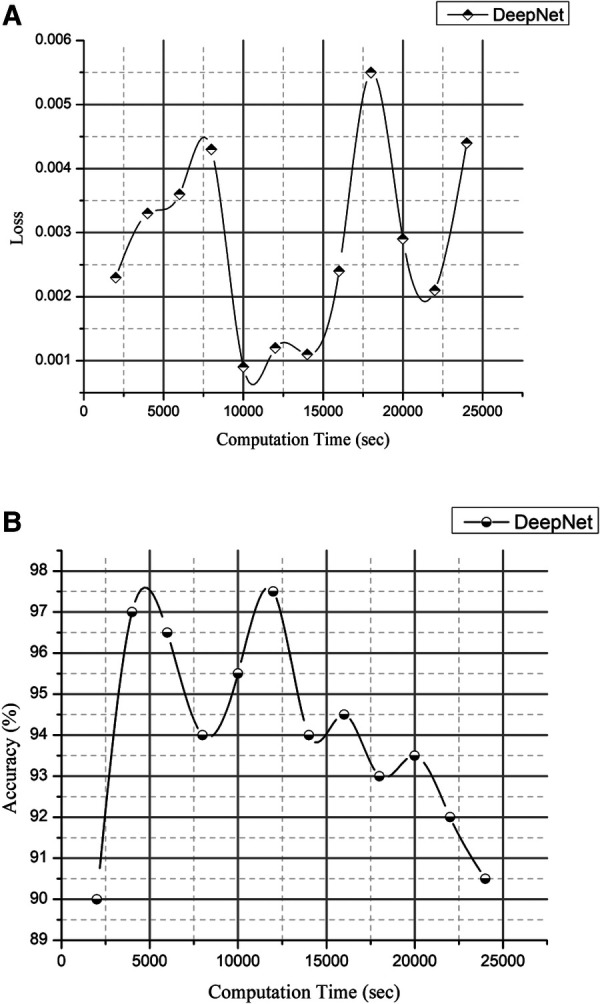
(**A**) computation time vs. loss and 1(**B**) computation time vs. accuracy.

The experimental study on the CT image collection includes measurements of computation time, losses, and accuracy. In addition to explaining lung cancer, the DeepNet model is evaluated using the Kaggle dataset for training and testing reasons for cancer segmentation and classification in MATLAB. In this case, samples of photographs are fed into a network model trained to detect cancer in pictures (both malignant and benign). Table 4 and Figures 12A,B show the outcomes of experimental research on a CT scan image collection for computation time, loss, and accuracy.

**Table 4:** Computation time verses loss and accuracy.

Computation Time (Sec)	Loss	Accuracy (%)
30	0.71	73.5
1,248	0.022	82.3
2,626	0.021	87.9
3,908	0.0032	90.2
4,930	0.0044	91.6
6,075	0.0011	92.3
7,142	0.0021	92
8,343	0.0055	92.5
9,689	0.0029	94
10,214	0.0024	94.5
11,692	0.0026	94.8
12,242	0.0011	94.9
13,743	0.0012	95
14,357	0.0013	95.1
15,820	0.0007	95.8
16,818	0.0036	96
17,965	0.0031	96.4
18,745	0.0043	97.5
19,810	0.0033	97.7
20,835	0.0023	98.1
21,870	0.0009	99.5

The loss and accuracy of the DeepNet for Computation time is shown in Figures 12A,B with the loss of 0.0007 and accuracy of 99.5. Compared to previous research articles, DeepNet's final result is the highest degree of accuracy obtained: 99.6% with a calculation duration of 45,141 s on a single-CPU workstation.

Figure 11 is a visual depiction of the accuracy percentages from the several studies included in the literature review. Figure 12 compares the computation time of the existing method with DeepNet. This results in improved classification accuracy, less loss and computation time that exceeds that of 3D LungNet (16) SegNet (17) MAU-Net (18), ResNet-34 model (21).

#### 4.3.3. Root mean square error (%)

**Figure 13:**
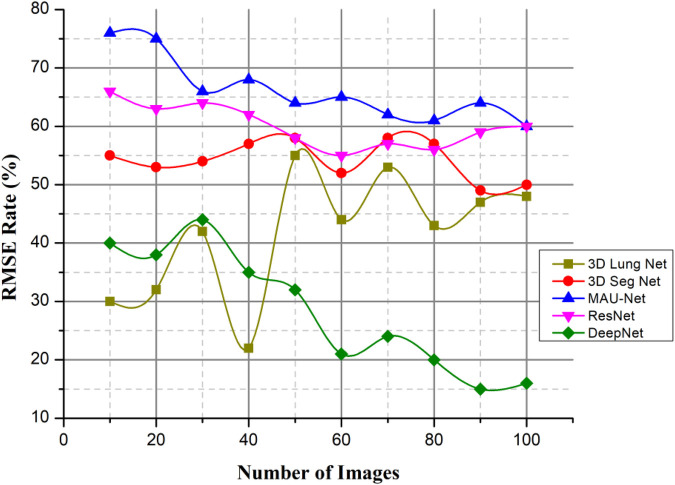
RMSE rate.

The author presented a technique to categorize lung nodules using CT scans. The lung segmentation would take place via background subtraction and a cuckoo search algorithm, and the image characteristics would then be retrieved. The collected characteristics are given for different classifiers, such as DeepNet. Afterward, the classifier determines and categorizes the photos as either benign or malignant. To locate lung nodules, the author suggests using the DeepNet classifier, which has an accuracy rate of around 99.6%. Figure 13 deliberates the RMSE Rate with low error based on the cuckoo search algorithm (CSA).

(15) $$\begin{array}{rl} & \mathrm{Imbalance}\;\mathrm{Coefficient}t_{L-R}\\ & \;=\frac{\;\mathrm{Tidal}\;\mathrm{ventilatio}n_{\mathrm{left}}-\mathrm{Tidal}\;\mathrm{ventilatio}n_{\mathrm{right}}\;}{\mathrm{Tidal}\;\mathrm{ventilatio}n_{\mathrm{left}}+\mathrm{Tidal}\;\mathrm{ventilatio}n_{\mathrm{right}}} \end{array}$$

This value is scaled such that it would fall somewhere between +1 (where the left lung is ventilated; $\mathrm{Tidal}\;\mathrm{ventilatio}n_{\mathrm{right}}=0$) and 1 (where the right lung is ventilated; $\mathrm{Tidal}\;\mathrm{ventilatio}n_{\mathrm{left}}=0$). Acute respiratory injury and baseline values for the imbalance coefficient $\mathrm{Imbalance}\;\mathrm{Coefficien}t_{L-R}$ were determined for each pixel within every location. This paper presents a model for classifying lung cancer nodules from CT scans. The model uses many classifiers to identify cancer, which increases the model's efficiency and, as a result, lowers the error rate.

#### 4.3.4. F1-score ratio (%)

Fusing deep learning concepts for lung nodule categorization is proposed as a solution to the issues of the field, such as the lengthy and difficult classification detection phase, low accuracy rate, and high false positive rate. The model's architecture is a convolutional network model with 50 layers, and it reconstructs the average global pooling layer, the FC layer, and the classification algorithm.

(16) $$F1-\mathrm{score}=\frac{T^{+}}{T^{+}+\frac{1}{2}(F^{+}+F^{-})}\;$$

**Figure 14:**
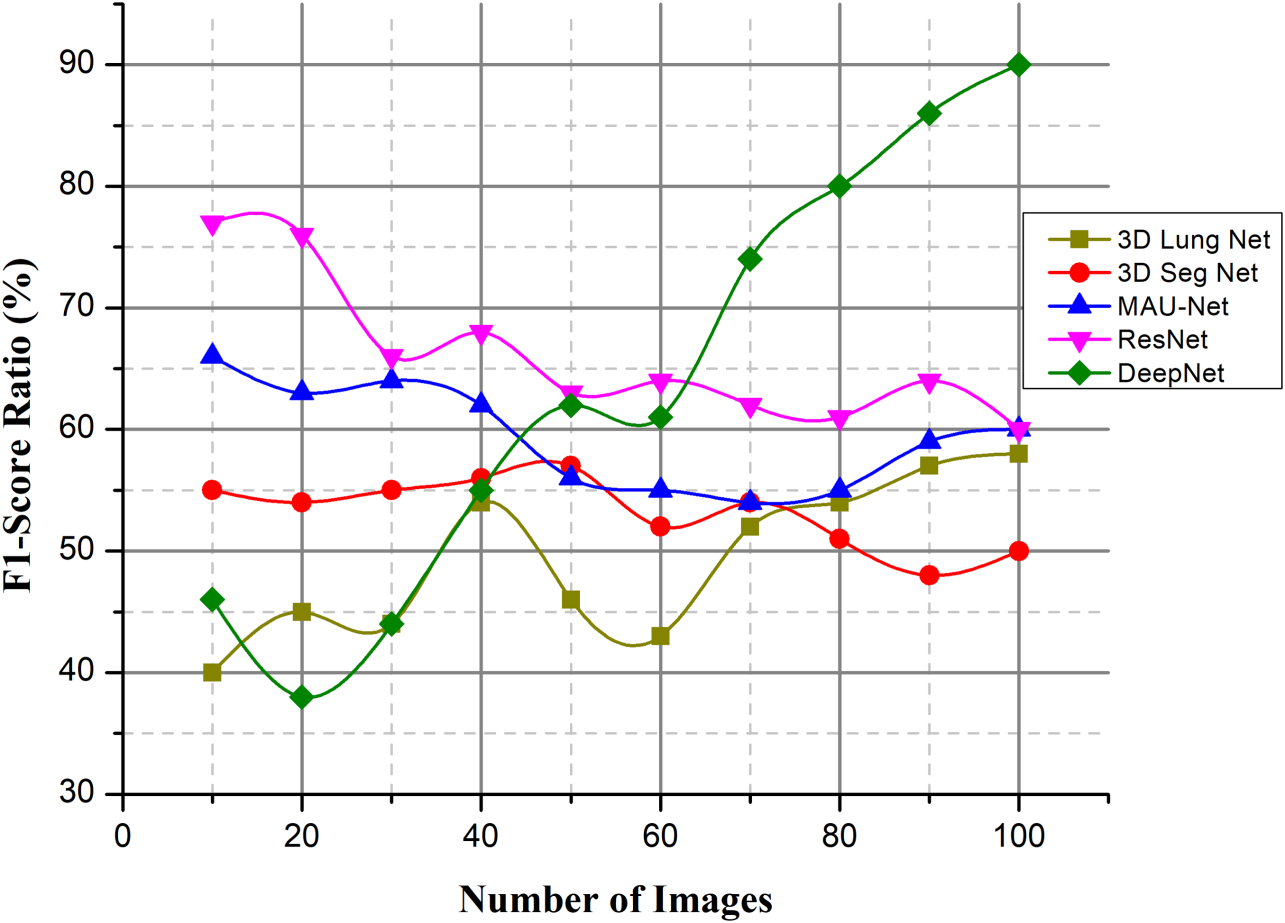
F1-score ratio (%).

As the abovementioned equation shows the F1-score value and Figure 14 when compared to other existing methods, DeepNet achieves a high value with the help of CSA. $T^{+}$ represent the total number of positive values, $F^{+}$ denote the false positive values, $F^{-}$ represent the false negative values. The experimental findings on the Kaggle dataset show that the DeepNet model outperforms the published results of all the other methods, including the neural network models and a typical ML algorithm, in terms of accuracy and F1 score. Comparing the suggested technique to those of existing classifiers, it emerges with the highest scores in terms of both accuracy and F1-score.

#### 4.3.5. Efficiency ratio (%)

The patient's diagnosis is confirmed alongside the formulation of a suitable treatment strategy when the nodule information (density, shape, and texture traits) is analyzed for the potential presence of malignancy. The process of recognizing the nodules is a difficult one. Inadequate professional expertise, distractions, or exhaustion when recording scans, among other factors, may weaken nodule detection, contributing to mischaracterizations of false positives using the available data. The DeepNet model needs to be highly sensitive while having a low number of false positives, a low installation cost, a low cost of maintenance tasks, vulnerability management assurance, and high levels of automation to improve efficiency. The lung CT images are obtained from the dataset. The image noise is removed using an approach called weighted histogram equalization.

**Figure 15:**
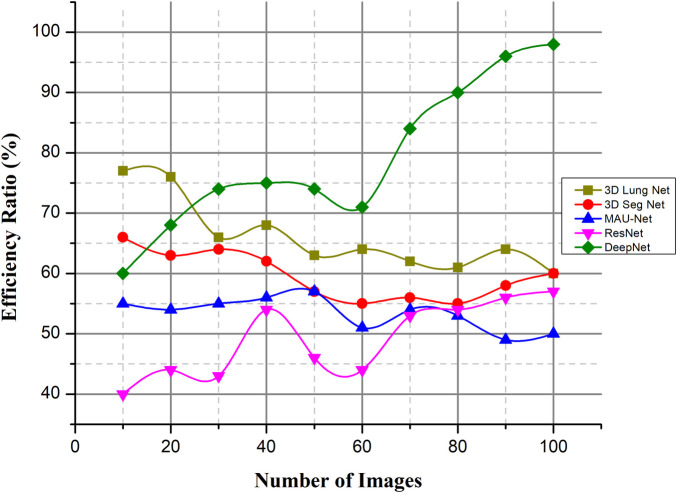
Efficiency ratio (%).

As illustrated in Figure 15, efficiency ratios are compared with the existing methods. This successfully removed the noise from the image, which improved the image quality. The cuckoo search algorithm is utilized to segregate the affected region. Several spectral characteristics may be extracted from the compressed area. These are investigated using a DeepNet to diagnose lung cancer.

## 5. Conclusion

Lung cancer is the deadliest type of cancer. Developing an automatic and reliable system to segment lung nodules from a CT image is a very practical tool in the healthcare industry. The DeepNet model detects and separates lung nodules in CT scans. The DeepNet model employs an encoder/decoder network to accomplish pixel-wise picture segmentation. With the help of 16 upsampling and deconvolution modules, the decoder network improves upon the output of an encoder network that uses a Visual Geometry Group (VGG-19) model as its foundational design. The structural design of the decoder can be trained to produce outputs of arbitrary resolution, depending on the size of the input scans. The encoder's fuzzy data is mapped and upsampled by the decoder's network. As a result, the network reuses the pooling indices of the encoder for segmentation, drastically reducing the total number of variables required for training. The cuckoo search algorithm is used to find the most relevant features. The DeepNet model is evaluated based on the real-world database known as The Cancer Imaging Archive (TCIA) dataset. It shows its efficacy by comparing its representation to other contemporary segmentation methods along a few key performance metrics. The empirical analysis shows that DeepNet significantly outperforms other prevalent segmentation algorithms with 0.962 ± 0.023% of volume error, 0.968 ± 0.011 of dice similarity coefficient, 0.856 ± 0.011 of Jaccard similarity index, and 0.045 ± 0.005 s average processing time. The accuracy ratio of our prosed method is 99.5%, 99.3%, and the loss is 0.008, 0.009 verses of epoch, iteration, and computation time. The overall efficiency ratio of our method is 98.7%, the F1-score ratio of 96.2% and the RMSE value is 0.0016 compared to other existing methods. Because of GPU memory space limitations, the pictures of this data were reduced to 120×120 pixels before our training step. In the future, this method would like to figure out what causes lung cancer in people and use that information to predict cancer. The DeepNet has highest efficiency ratio, F1-score with average processing time, less error rate. F1-Score of DeepNet is high with less RMSE value and loss ratio. The DeepNet method is compared with the loss and accuracy in terms of epoch, iteration and computation time. The segmentation algorithm gives better result in the form of volume error, dice similarity coefficient.

## Data Availability

The original contributions presented in the study are included in the article/Supplementary Material, further inquiries can be directed to the corresponding author.
